# National BSUG audit of stress urinary incontinence surgery in England

**DOI:** 10.1007/s00192-018-3705-4

**Published:** 2018-07-11

**Authors:** Swati Jha, Tim Hillard, Ash Monga, Jonathan Duckett

**Affiliations:** 1 Department of Urogynaecology, Sheffield Teaching Hospitals NHS Foundation Trust, Jessop Wing, Tree Root Walk, Sheffield, S10 2SF UK; 2 Department of Obstetrics and Gynaecology, Poole Hospital NHS Foundation Trust, Longfleet Road, Poole, Dorset BH15 2JB UK; 3 Urogynaecology Unit, University Hospitals Southampton NHS Trust, Tremona Road, Southampton, SO16 6UY UK; 4 Department of Obstetrics and Gynaecology, Medway Hospital, Windmill Rd, Gillingham, Kent ME7 5NY UK

**Keywords:** Stress urinary incontinence, Audit, Midurethral sling, Colposuspension, Urethral bulking, Autologous fascial sling

## Abstract

**Introduction and hypothesis:**

The aim of the British Society of Urogynaecology (BSUG) 2013 audit for stress urinary incontinence (SUI) surgery was to conduct a national clinical audit looking at the intra- and postoperative complications and provide outcomes for these procedures. This audit was supported by the Healthcare Quality Improvement Partnership (HQIP) and National Health Service (NHS) England.

**Methods:**

Data were collected for all continence procedures performed in 2013 through the BSUG database. All clinicians in England performing SUI surgery were invited to submit data to a central database. Outcomes data for the different continence procedures were collected and included intraoperative and postoperative complications and the change in continence scores at postoperative follow-up Changing trends in stress incontinence surgery were also assessed.

**Results:**

We recorded 4993 urinary incontinence procedures from 177 consultants at 110 centres in England: 94.6% were midurethral slings; 86.7% (4331) were submitted by BSUG members with the remaining 13.3% submitted by non-BSUG members. Postoperative follow-up data were available for 3983 (80%) patients: 92.3% (3676) were very much better/much better postoperatively, and 4806 (96.3%) proceeded with no reported complications. There were 187 cases (3.7%) in which a perioperative complication was recorded. Pain persisting >30 days was reported in 1.9% of all patients.

**Conclusions:**

Surgery for SUI has good outcomes in the short term. Midurethral synthetic slings have been shown to be safe and effective as a treatment option, with >90% being very much/much better at their postoperative follow-up.

## Introduction

In 2012, concerns about the use of vaginal mesh products in incontinence and prolapse surgery were raised in the media and subsequently in the Scottish Parliament and by National Health Service (NHS) England. The British Society of Urogynaecology (BSUG) collaborated with a number of organisations, including NHS England, the Royal College of Obstetricians and Gynaecologists (RCOG) and the British Association of Urological Surgeons (BAUS), and wrote to all NHS trusts to address these concerns. This highlighted the lack of available outcome data for individual surgeons performing continence surgery. Although use of the BSUG database was well established in many surgeons’ clinical practice, not all surgeons performing continence surgery were entering their data on the database; many were not BSUG members, and some were urological surgeons. To help address this shortfall of information, NHS England, through the Healthcare Quality Improvement Partnership (HQIP), wished to support a national audit of stress incontinence surgery through the Consultant Outcomes Publication (COP). Professor Sir Bruce Keogh, NHS Medical Director at the time, wrote to all medical directors to this effect. HQIP commissioned BSUG to lead this audit, and all surgeons, both gynaecologists and urologists, carrying out stress incontinence surgery in England were expected to retrospectively produce their personal results for the period 1 January 2013 to 31 December 2013, which would be analysed and published. This was mandated only in England. Members in Wales, Northern Ireland and Scotland were not required to take part in this process but were invited to enter their data voluntarily if they so wished.

HQIP was established in April 2008. The main aim was to promote quality in healthcare particularly aimed at increasing the impact that clinical audit has on healthcare quality improvement. They are an independent organisation led by the Academy of Medical Royal Colleges, The Royal College of Nursing as well as National Voices. They commission, manage and support national and local programmes of quality improvement.

The aim of the audit for stress urinary incontinence (SUI) surgery was to conduct a national clinical audit of intra- and postoperative complications and provide outcomes for these procedures. Though individual outcomes for clinicians were collected, assessing that data is out with the remit of this study.

## Methods

An email was circulated by the executives of BSUG at that time informing the membership of the intentions to complete this audit through the BSUG database. An email was also sent to medical directors of each NHS trust informing them of this audit. This information was relayed by the governance leads to all gynaecologists/urologists performing continence surgery. An additional tab was created on the BSUG database that covered all questions relating to this audit. For BSUG members already using the database, there was little additional work, as the database automatically populated the relevant audit sections. They were, however, asked to check their data entries to ensure it was accurate and complete. For BSUG members not using the database, restricted access was granted to allow retrospective data entry on the audit page. Non-BSUG members undertaking continence surgery were given access to the audit section of the database to allow registration and data submission for the purposes of this audit.

The analysis included an assessment of all procedures undertaken for urinary incontinence (UI). The grade of operator, frequency with which urodynamics/cystometry was performed preoperatively and concomitant procedures performed were assessed. The risk of complications (intraoperative and postoperative) were broken down by procedure and included intraoperative complications and the need for catheterisation >10 days, readmission to hospital within 30 days, graft complications, pain persisting beyond 30 days and the Patient Global Impression of Improvement (PGI-I) index were also calculated. We also analysed the total number of these cases performed in 2017 and compared trends in type of surgery being performed with this audit data. All data were analysed using SPSS 23 (Released 2015; IBM SPSS Statistics for Windows, Version 23.0. Armonk, NY: IBM Corp). Simple proportions were presented as percentages, mean and ranges were performed in quantitative continuous data analysis, medians and interquartile range (IQR) in quantitative discrete data, and qualitative variables summarised using absolute numbers and percentages.

## Results

The audit of 2013 recorded 4993 cases input through the BSUG data base. Procedures were recorded from 177 consultants at 110 centres. Median number of cases per centre was 35 (range 1–174) and per consultant was 22 (range 1–173); 90.3% (4507) of procedures were performed for primary SUI and 7.2% (360) for recurrent SUI. The average age of patients undergoing surgery was 52.4 (18–95) years. Breakdown by procedure is shown in Table 1. Seventeen cases of intravesical botulinum were recorded and were thus excluded; in a further 48 cases, the procedure was not recorded. Synthetic midurethral slings (MUS) accounted for 94.6% of recorded procedures (4928). Retropubic midurethral synthetic tape was the most commonly performed procedure, accounting for 3092 (62.7%) of the total number of cases, followed by the obturator midurethral synthetic tape, which accounted for 1573 (31.9%). Open/ laparoscopic colposuspension and autologous fascial slings (AFS) accounted for a very small proportion of cases, at 0.8% (40 cases).

**Table 1:** Procedures assessed in the Healthcare Quality Improvement Partnership (HQUIP) audit

Procedure performed	No. HQIP audit 2013 (4928)	No. HQIP audit 2017
Retropubic midurethral sling	3092	1852
Obturator midurethral sling	1573	533
Single-incision sling	113	25
Bladder-neck injection	86	713
Open colposuspension	32	126
Laparoscopic colposuspension	8	85
Autologous fascial sling	16	61
Remeex (adjustable sling)	3	0
Diverticulum	2	x
Stamey	1	x
Anterior repair	1	x
Fistula	1	x

Pelvic floor exercises were documented in 76% of patients, and preoperative urodynamics were performed in 90%. Most patients were seen by consultants (84.7%) who is a senior hospital-based surgeon who has completed specialist training and accepts ultimate responsibility for the care of the patients referred to them. Most patients had no concomitant surgery (2998; 60%). However, when additional procedures were performed, anterior (527) and posterior repair (518) either in isolation or in conjunction with other procedures were the most commonly performed concurrently. Complications by procedure are shown in Table 2. One hundred and eighty-seven (3.7%) patients had a reported intraoperative complication or a return to theatre in the postoperative period. Reasons included bladder injury, urethral injury, vaginal buttonholing, vascular injury, nerve damage or the need for a blood transfusion during hospital stay. There were no ureteric or bowel injuries. The risk of bladder injury with a retropubic midurethral synthetic tape was 3.6% (consultants 1.8%; other operators 5.4%) but were reported with obturator tapes also, albeit in very small numbers (0.2%). Major complications, such as vascular injury, nerve damage and thromboembolism were rarely reported (<0.1%) for any procedure.

**Table 2:** Complications

Procedure (n)	Bladder injury *n* (%)	Urethral injury *n* (%)	Vaginal buttonholing *n* (%)	Vascular injury *n* (%)	Nerve damage *n* (%)	Blood transfusion *n* (%)	ThromboEmbolism *n* (%)	Return to theatre *n* (%)	Catheterisation >10 days *n* (%)	Readmission within 30 days *n* (%)
Retropubic synthetic tape (3092)	113 (3.6)	2 (<0.1)	17 (0.5)	2 (<0.1)	1 (<0.1)	2 (<0.1)	3 (<0.1)	13 (0.4)	99 (3.2)	108 (3.4)
Obturator synthetic tape (1573)	4 (0.2)	0	14 (0.9)	0	0	1 (<0.1)	0	10 (0.6)	19 (1.2)	25 (1.6)
Open colposuspension (32)	1 (3.1)	0	0	0	0	1 (3.1)	0	1 (3.1)	5 (15)	3 (9.3)
Laparoscopic colposuspension (8)	0	0	0	0	0	0	0	0	0	0
Autologous fascial sing (16)	0	0	0	0	0	0	0	0	7 (43%)	1 (6.2)
Single-incision sling (113)	0	0	0	0	0	0	0	0	1 (0.8)	5 (4.4)
Bladder-neck injection (86)	0	0	0	0	0	0	0	1 (1.1)	1 (1.1)	1 (1.1)
Remeex (3)	1 (33)	0	0	0	0	0	0	1	0	1 (33)

The overall risk of catheterisation required after 10 days was significantly greater with an AFS (43%) and colposuspension (15%) when compared with a midurethral synthetic tape. Postoperative follow-up data were available for 3983 (80%) patients. Of these, 3676 (92.3%) patients themselves reported they were very much better/much better at their follow-up visit. Results for individual procedures are shown in Table 3. The follow-up visit was undertaken from 6 weeks to 12 months after surgery, with most being at 3 months (38%). Pain persisting >30 days was reported in 1.9% of all patients undergoing MUS but similar in retropubic and obturator MUS. Pain was significantly higher in those undergoing an AFS (12.5%) compared with a midurethral synthetic tape (1.9%), although numbers were small (*p* < 0.01).

**Table 3:** Outcomes by Patient Global Impression of Improvement (PGI-I) index, including pain

Procedure (n)	Very much better/much better/a little better *n* (%)	A little better/no change *n* (%)	A little worse/much worse/ very much worse *n* (%)	Pain after 30 days *n* (%)
Retropubic synthetic tape (2391)	2213 (92.5)	160 (6.7)	18 (0.7)	56 (1.8)
Obturator synthetic tape (1329)	1242 (93.4)	80 (6.0)	7 (0.5)	33 (2)
Open colposuspension (27)	25 (92.6)	1 (3.7)	1 (3.7)	0
Laparoscopic colposuspension (6)	6 (100)	0	0	0
Autologous fascial sing (14)	13 (92.8)	1 (7.1)	0	2 (12.5)
Single-incision sling (94)	89 (94.6)	4 (4.3)	1 (1.1)	3 (2.6)
Bladder-neck injection (73)	45 (61.6)	27 (36.9)	1 (1.3)	0
Remeex (3)	2 (67)	1 (33)	0	0

Graft complications were reported in 1.3% (39) of retropubic synthetic tapes, 1.3% (22) of obturator synthetic tapes and 0.8% (1) of single-incision synthetic tapes within the follow-up period. Two deaths occurred during the study period (one following a colposuspension and the other following an obturator synthetic tape), but it was difficult to establish causation. A considerable reduction in the number of synthetic MUS insertions was reported that halved the number of these procedures by 2017 (4781–2410). Whilst bladder-neck injections saw an eightfold increase (86–713), colposuspensions (combined open and laparoscopic) increased fivefold (40–211) and AFS fourfold (16–61) (Table 1). MUS continue to account for >70% of SUI procedures.

## Discussion

SUI has good outcomes in the short term. MUS have been shown to be a safe and effective treatment option, with >90% being very much/much better at postoperative follow-up. Severe complications were rare, but small numbers for operations other than synthetic tapes made comparisons difficult. There was a trend towards more complications (return to theatre, catheterisation >10 days, readmission, persisting pain >30 days) in colposuspension and AFS. Pain was seen after 30 days in 1.9% of patients undergoing an MUS.

There are several reports from other registries, including some stating their early results (Austrian registry) [1] then going on to report large cohorts with longer-term follow-up data [2]. Other countries, including The Netherlands [3, 4], France [5], Norway [6] and Denmark [7], have published single operation results or reviews of their whole database. In the UK, there have been previous publications on various aspects of the BSUG database [8]. The work we present here is different in that it describes the activity and risks of the different SUI procedures over the period of 1 year. This allows trend mapping and risk and complication identification of individual operations. Surgeons need comparative data when counselling patients regarding risks and benefits of different surgical procedures. Our report enables comparisons of different procedures and their complications, and analyses will be stronger when more data is available for operations performed less commonly. Whilst using a whole database to identify a single operation over a long time period is undoubtedly useful, it includes surgeon learning curve of that procedure early in the period. The initial aim of this audit was to provide yearly outcome data for individual surgeons and analyse outcomes of procedures for SUI, specifically, assessment of complications such as pain. Providing COP data, however, is a costly and labour-intensive process, and providing individual consultant outcomes would require a significant investment from government to provide this information on an annual basis.

Other large data sets have been acquired using routinely collected data such as Hospital Episode Statistics (HES data) in England [9]. According to the recent NHS digital retrospective review of mesh procedures, in 2013–2014, 11,786 synthetic tape surgeries were performed [10]. This data is dependent on the accuracy of coding and does not provide any information regarding patient outcome and is of limited value in a clinical context. In addition, it is difficult to accurately compare that data with this audit, as data captured through HES is from April through March of the subsequent year, whereas our data was from January through December. Individual complications are difficult to identify, but longer-term data sets are complimentary to the research described in this paper. Such studies reported a 9.8% complication rate after MUS surgery but did not define what those complications were. Many were related to voiding, which is a recognised consequence of any continence surgery rather than a complication. The type of data presented in our paper provides some of the information missing from HES data sets.

Large clinical data sets are useful for providing insight into clinical practice and real-life outcomes, but they have limitations. It has been recommended that all continence surgeons in England use the BSUG database [11], but it is difficult to mandate its use through the current commissioning process for healthcare. It is difficult to be precise, but this audit of gynaecologists collected almost 5000 cases from (probably) 8000 cases performed in that year by gynaecologists. The follow-up of 80% was good. There is a risk or reporting bias where users do not adequately record poor outcomes or complications. Some users may not report all cases or may have insufficient resources to chase full follow-up data. These are issues common to all databases. Variable follow-up points for outcomes may affect results. Long-term complications may be missed with this type of yearly audit. The reporting of graft complications in particular requires long-term follow-up. Rates reported in this audit, at 1.3%, represent early complications that are likely to be a consequence of poor healing of vaginal mucosa and midline exposure rather than mesh migration into adjacent organs, which may be detected with longer-term follow-up. In 2017, the BSUG database was redesigned to capture data relating to complications such as mesh removal. Pain after 30 days was uncommon after MUS but was seen in 1.9% of patients. Pain severity was not documented, and we do not know if any further treatment was required for pain or outcomes of any treatment.

Data presented in this paper provide information on a large cohort of MUS synthetic tapes and suggest good success according to the PGI-I scale and a low risk of complications. Major complications were uncommon. Other operations, such as fascial slings and colposuspension, may have higher complication rates, but more data is needed to establish this.

The BSUG is investigating the feasibility of analysing patients in the 2013 audit at the current time to see whether more information can be obtained on longer-term outcomes related to the use of synthetic mesh as well as failure and recurrence rates of SUI surgery.
